# Three Decades of Success for *Clinical Neuroradiology*

**DOI:** 10.1007/s00062-021-01090-6

**Published:** 2021-09-24

**Authors:** László Solymosi

**Affiliations:** Dept. of Neuroradiology, Universitätsklinikum Würzburg, Würzburg, Germany

*Clinical Neuroradiology* was founded 30 years ago by the German Society for Neuroradiology with the aim of promoting “increased cooperation between clinically and diagnostically active physicians” [1]. The first editors were Professors Maschallah Nadjmi, Hans Hacker, Klaus Sartor and Karsten Voigt. Later, the Austrian and Swiss societies also joined and the journal became the official journal of the German-speaking neuroradiological societies.

In the last 30 years we have seen an enormous development in neuroradiology. As the best example, 30 years ago the first aneurysm was treated with detachable coils, which has become the standard therapy today. Parallel to this development of the subject, the number of “full-time” neuroradiologists also grew, and in Germany, with a few exceptions, independent clinics and chairs for neuroradiology were established at universities. Today, the German Society for Neuroradiology is the largest national neuroradiological society in Europe with over 1000 members.

**Fig. 1:**
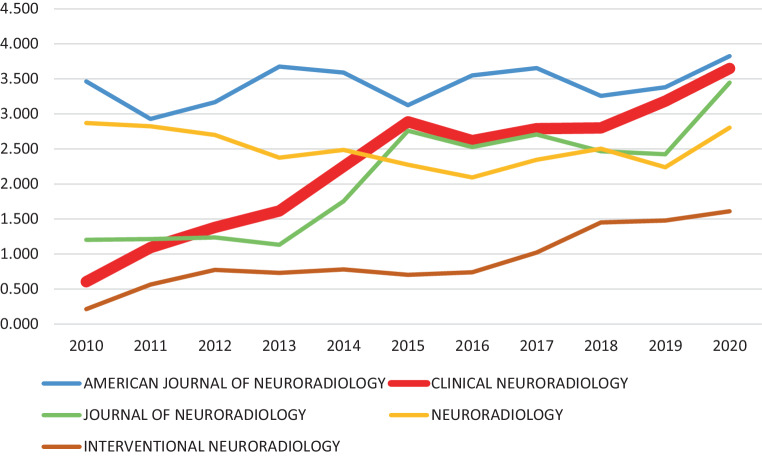
Development of the impact factor in recent years (data from Clarivate [3])

The enormous development in neuroradiology is reflected in the development of *Clinical Neuroradiology*: The number of papers submitted has multiplied, the issues have become thicker, and the quality of the published papers has increased continuously. Ten years ago, we were able to report that the journal received an impact factor for the first time [2]. A testimony to the journal’s success, the impact factor has risen continuously in recent years (Fig. 1). The current impact factor (2020) lies at 3.649.

Although the increase in the impact factor by itself is an important quality feature, one must also consider the ranking in the two categories, “Clinical Neurology” and “Radiology, Nuclear Medicine & Medical Imaging”. In these categories, we have a stable position in the upper half.

The increase of the impact factor, the rising number of submissions and also the continuous growth of articles accessed and downloaded show that *Clinical Neuroradiology* has become internationally recognized and is now one of the most respected neuroradiology journals by both authors and readers. We have taken care to ensure that the quality of the contributions remains the most important criterion for publication and will remain so in the future. The topics of the papers submitted correspond to the current scientific activities of the neuroradiological community and are independent from the editorial board.

However, we still see opportunities to improve and for this we still need your support. Please continue to read *Clinical Neuroradiology* and consider the published papers in your research. We on the Editorial Board assure a fair, rapid and robust peer review process. We are also available to you as contact persons for any questions or concerns at any time.

László Solymosi

Editor-in-Chief
